# Chia (*Salvia hispanica*)-supplemented diet ameliorates non-alcoholic fatty liver disease and its metabolic abnormalities in humans

**DOI:** 10.1186/s12944-020-01283-x

**Published:** 2020-05-19

**Authors:** Aida Medina-Urrutia, Angel R. Lopez-Uribe, Mohamed El Hafidi, Maria del Carmen González-Salazar, Rosalinda Posadas-Sánchez, Esteban Jorge-Galarza, Leonardo del Valle-Mondragón, Juan G. Juárez-Rojas

**Affiliations:** 1 Departamento de Endocrinología, Juan Badiano 1, Col. Sección XVI, Tlalpan, Mexico City, Mexico; 2 Departamento de Biomedicina Cardiovascular, Juan Badiano 1, Col. Sección XVI, Tlalpan, Mexico City, Mexico; 3 Departamento de Farmacología, Juan Badiano 1, Col. Sección XVI, Tlalpan, Mexico City, Mexico

**Keywords:** Non-alcoholic fatty liver disease, Chia, Visceral abdominal fat, Cardiometabolic risk

## Abstract

**Background:**

Non-alcoholic fatty liver disease (NAFLD) is a public health problem lacking an approved pharmacological treatment. Omega-3 fatty acids have shown to reverse NAFLD. Chia is a seed rich in α-linolenic acid (ALA), antioxidants, and fiber; therefore, it could be useful to treat NAFLD.

**Methods:**

In a single arm experimental design study, the effect of 25 g/day of milled chia was assessed in 25 patients with NAFLD. After two weeks of dietary stabilization (basal condition) and eight weeks of a chia-supplemented isocaloric diet, liver:spleen attenuation index and visceral abdominal fat (VAF) were measured by computed tomography. Lipids, lipoproteins, free fatty acids (FFA), and ALA plasma concentrations were also determined.

**Results:**

Dietary chia supplementation induced an increase in plasma ALA concentration (75%) and dietary fiber (55%) consumption. After chia supplementation, VAF (9%), body weight (1.4%), total cholesterol (2.5%), non-high density lipoprotein cholesterol (3.2%), and circulating FFA (8%) decreased. Furthermore, NAFLD regressed in 52% of the treated patients (*P* < 0.05 for all).

**Conclusions:**

The results of the present study show that 25 g/day of milled chia ameliorates NAFLD. Chia is an accessible vegetal source of omega-3 fatty acids, antioxidants, and fiber, which could have the potential to prevent metabolic abnormalities in NAFLD patients. Considering that there is no pharmacological treatment approved for NAFLD, the findings of the present study suggest that a chia-supplemented diet could be an innovative alternative to control this disease.

**Retrospectively registered:**

https://clinicaltrials.gov/show/NCT03942822

## Introduction

Parallel to the obesity epidemic, non-alcoholic fatty liver disease (NAFLD) prevalence has markedly increased during the last years [1]. Recent epidemiological studies have found that one of three adults has NAFLD, which has been associated with a cluster of metabolic abnormalities [1, 2]. As noted, poor-quality diets characterized by high fructose content and deficient omega-3 fatty acids consumption, scarce physical activity, excess in visceral abdominal fat (VAF), insulin resistance, and genetic susceptibility have shown to be relevant determinants for this hepatic disorder [2–4]. It has been observed that type 2 diabetes mellitus (T2DM) and coronary artery disease (CAD) are the most frequent complications of NAFLD; nonetheless, it can also progress to cirrhosis and hepatic carcinoma [1, 2]. Hence, NAFLD is considered a multisystemic disease and a public health problem [1].

Although no drug has been specifically approved for the treatment of NAFLD, recent studies indicate that dietary supplementation with marine-origin omega-3 fatty acids (eicosapentaenoic acid [EPA]/docosahexaenoic acid [DHA]) and the Mediterranean-style diet are useful to treat NAFLD [4, 5]. EPA and DHA reduce the intrahepatic fat content and improve the metabolic profile observed in these patients, even in the absence of caloric restriction diets [5]. However, due to the socioeconomic and sociocultural characteristics of some populations, the consumption of these foods is complicated, increasing the interest in searching for alternatives of omega-3 fatty acids from vegetal sources [6].

Therapies that focus on the use of functional foods, rich in a variety of phytochemicals, mono/polyunsaturated fatty acids, antioxidants, minerals, and fiber, have shown antioxidant, anti-inflammatory, and lipid-lowering effects [7, 8], which could be useful in patients with NAFLD [6]. Chia seed (*Salvia hispanica*) is the richest vegetal source of omega 3-fatty acids, antioxidants, and fiber [9]. Although some animal models have suggested that chia could be used as an alternative to reduce intrahepatic fat content [10, 11], its effect on NAFLD patients has not been studied yet. Hence, the objective of the present study was to analyze whether the consumption of an isocaloric diet supplemented with chia could ameliorate NAFLD, VAF, and metabolic abnormalities in NAFLD patients.

## Materials and methods

### Participants

**Fig. 1:**
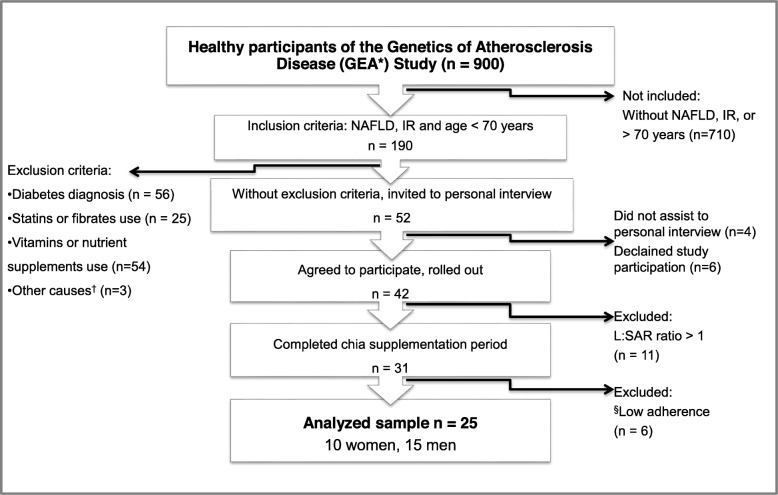
Sample selection. *GEA for its initials in Spanish (Genética de la Enfermedad Aterosclerosa). NAFLD (non-alcoholic fatty liver disease), IR (insulin resistance: HOMA-IR < 3.7 in men and 3.4 in women) [10], L:SAR (liver:spleen attenuation ratio), ALA (alpha linolenic acid), †Other causes (psychiatric disorders, significant weight change), §ALA plasma increase < 30%

Participants were chosen from the control group of the Genetics of Atherosclerotic Disease (GEA, for its initials in Spanish) study, performed at the *Instituto Nacional de Cardiología Ignacio Chávez* in Mexico City, Mexico (Fig. 1). Eligible subjects were those younger than 70-years (30–69 years) with NAFLD diagnosis confirmed by computed tomography (CT) imaging and with insulin resistance assessed by the homeostatic model assessment insulin resistance index (HOMA-IR) [12]. Exclusion criteria were previous diabetes diagnosis, use of hypoglycemic or hypolipidemic medications, unstable body weight (variation > 5% within the preceding 3-month period), consumption of vitamins, herbal or food supplements, gastrointestinal, renal, or hepatic diseases, and the presence of eating, psychiatric or cognitive disorders that would hurdle the understanding of the study instructions and their compliance. Candidates that accepted to participate in the study signed a written informed consent form prior to completing any assessment.

The experimental protocol was conducted according to the ethical guidelines of the 1975 Declaration of Helsinki, it was approved by the Research and Ethics Committee of the *Instituto Nacional de Cardiología Ignacio Chavez* (No. 16–980), and was retrospectively registered at clinical trials.gov (NCT03942822).

### Nutritional intervention and food intake evaluation

To know the current eating habits, including total energy, macronutrients and fiber intake, 24-h dietary recalls were applied on the first visit by a trained nutritionist. As previously recommended, two weekdays and one day of the weekend were considered for the recalls [13]. To standardize macronutrient dietary composition, two weeks before starting chia supplementation, each patient was interviewed by the nutritionist aimed at designing and explaining his/her isocaloric diet (30% of total energy as fat, 15% of energy as protein, and 55% of energy as carbohydrate) to be followed during the study. After dietary standardization, each patient was provided monthly with 30 sachets of 25 g of chia seeds. In the first month, a 4-oz glass jar blender (Oster® Classic Series Kitchen) was provided to mill chia seeds, and the patients were instructed to mill one sachet per day, pointing out the relevance of consuming the milled seed from breakfast through lunch, but always before 6:00 p.m. It was recommended to intake chia with water, salads, or cold dishes. To foster treatment adherence and record adverse events (appetite loss, constipation, diarrhea, flatulence and nausea, allergy or chia intolerance), patients were contacted once a week during the intervention. Since ALA is an essential fatty acid that can only be given through the diet, a higher than 30% increase in plasma ALA concentration was considered an indicator of appropriate compliance of chia consumption [14]. In addition, in patients who had an increase in ALA equal or higher than 30%, the adherence to seed consumption was calculated as the median of the empty chia sachets returned by the patients. Thirty empty sachets were considered as 100% compliance. Anthropometric, diet evaluation, laboratory test, and computed tomography studies were made after dietary standardization (basal condition), and after 8 weeks of chia supplementation. Patients were advised to maintain dietary recommendations and their usual level of physical activity throughout the study.

### Anthropometric evaluation

Anthropometric measurements were obtained according to the International Society for the Advancement of Kinanthropometry (ISAK) regulations, by a certified nutritionist. Weight and height of the patients were recorded before 8:00 a.m. in fasting conditions; participants were required to remove shoes and all outer cloths and heavy pocket items. A digital scale (Soehnle Professional Design 7830, Leifheit, Nassau, Germany), and a SECA 220 wall stadiometer (seca GMBH and Co. KG, Hamburg, Germany) were used, with an accuracy of 0.1 kg and 0.1 cm, respectively. Body mass index (BMI) was calculated as weight (kg)/height (m^2^). Waist circumference was measured with a non-stretch tape (Lufkin W606PM 6 mm × 2 m, Zapopan, Jal. Mexico). The measurement was made in duplicate, with the patient without clothes around the waist, at the midway between the lowest rib and the iliac crest, measures were averaged; however, a third measurement was taken if they differed more than 0.5 cm.

### Laboratory tests

After 10-h fasting and 20 min in sitting position, venous blood was collected in assay tubes without anticoagulant and in tubes with K2-EDTA (1.8 mg/mL). Glucose, total cholesterol (TC), triglyceride (TG), high-density lipoprotein cholesterol (HDL-C), alanine aminotransferase (ALT), aspartate aminotransferase (AST), gamma glutamyl transpeptidase (GGT), and uric acid (UA) concentrations were determined using direct standard enzymatic colorimetric methods (Roche Diagnostics, Mannheim, Germany), in a COBAS c311 analyzer. Low-density lipoprotein cholesterol (LDL-C) concentration was estimated using the DeLong formula [15]. The reproducibility and precision of these determinations were assessed by the Center for Disease Control and Prevention Lipids Standardization Program (LSP-CDC, Atlanta, GA, USA). Free fatty acids (FFA) were determined in plasma by an enzymatic-colorimetric assay (Wako Diagnostics, Chuo-Ku Osaka, Japan).

Liver enzyme values were considered high when ALT, AST, or GGT were ≥ percentile 75 (ALT: 23 U/L and 30 U/L, AST: 27 U/L and 29 U/L, GGT: 21 U/L and 28 U/L, for women and men; respectively). These cut-off values were obtained from 101 men and 180 women in the GEA study, without obesity and normal lipid, glucose, and blood pressure values.

### Plasma total fatty acid (TFA) analysis

To 100 μL of plasma, 50 μL of a solution of heptadecanoic acid (C17:0, Sigma Co., St. Louis, MO, USA) (1 mg/mL) in chloroform was added as an internal standard. All solvents and chemicals were analytical grade from J. T Baker (Avantor Performance Materials, Central Valley, PA, USA). Plasma lipids were subsequently extracted three times with a chloroform:methanol (1:2 *v/v*) mixture, by vigorous vortexing for 1 min, according to the Folch’s method [16]. After centrifugation, the organic layer was collected, combined, and the solvent evaporated at 40 °C under a nitrogen stream. Fatty acid transmethylation was immediately carried out at 80 °C, in a 2-mL mixture of methanol: H_2_SO_4_ (2%). Fatty acid methyl esters were extracted three times with 2 mL of n-hexane, which was separated and dried under nitrogen. The dry residue was dissolved in 50 μL of n-hexane, and 1 μL was analyzed in a Shimadzu GC-8A gas chromatograph equipped with a flame ionization detector (Shimadzu, Kyoto, Japan) and an SP2330 capillary column of 25 m length and 0.25 mm internal diameter (SUPELCO, Bellefonte, PA, USA). Fatty-acid peaks were identified by using the Supelco 37 component FAME Mix (CRM47885, SUPELCO, Bellefonte, PA, USA). A plasma control sample was run in each extraction assay; ALA inter-assay coefficient variation was lower than 13%.

### Chia’s fatty acid composition

The chia seeds were milled and analyzed for fatty acid content according to the AOAC [17] procedures, modified in our laboratory. Briefly, 100 mg of the milled seeds in the presence of 100 μg of heptadecanoic acid (C17:0), as internal standard, were stirred in 2 mL of a mixture of chloroform/methanol (2:1, *v/v*), containing 0.002% of butylated hydroxytoluene (BHT) as antioxidant, in an ice bed overnight. At the end of this period, 1 mL of NaCl solution (0.8%) was added and vortexed for 1 min; the solvent was decanted and collected. This step was repeated twice. The organic layer was dehydrated with anhydride sodium sulfate, filtered and evaporated under a gentle stream of nitrogen. Fatty acids of the residue were transesterified to their corresponding methyl ester and analyzed by gas chromatography as described above.

### Computed tomography study

Computed tomography is a validated method for measuring VAF [18] and evaluating NAFLD [19]. In the present study, these measurements were obtained using a 64-slice scanner (Somatom Cardiac Sensation 64, Forchheim, Bavaria, Germany). To determine the liver:spleen attenuation ratio (L:SAR), a single-slice CT-scan was obtained at the level of T11–T12 or T12–L1. Fatty liver was defined as a liver/spleen attenuation ratio lower than 1.0, which has been proposed as threshold for detecting moderate or severe steatosis (≥30%) by histology [19]. To calculate the amount of total abdominal fat (TAF) and VAF, a single-slice scan was performed at the level of L4–L5 and the area was expressed in square centimeters (cm^2^). Subcutaneous abdominal fat (SAF) was calculated by subtracting the VAF from the TAF area.

### Statistical analysis

Data are presented as mean ± standard deviation, median (interquartile range) or prevalence. Comparisons were made by paired Student’s t, Wilcoxon matched-pairs signed-rank or Chi-squared tests, as appropriate. *P* values < 0.05 were considered statistically significant. All the analyses were performed using SPSS for Windows (version 15.0; SPSS Chicago, II, USA).

## Results

The study included 25 NAFLD patients; general characteristics are shown in Table 1. All patients concluded satisfactorily 8 weeks of chia intervention at 25 g/day, with a mean adherence of 93 ± 8%. The fatty acid characterization of chia revealed that ALA (65%), linoleic (20%), oleic (6%), palmitic (6%), and stearic (3%) acids were the main components of the seed. After chia supplementation, an increase of plasma ALA (75% [40–125%]) and higher dietary fiber consumption (55% [17–92%]) was recorded (Table 2). At the beginning of the study, four subjects reported modest and transient gastrointestinal distress that did not merit their withdrawal from the study.

**Table 1:** General characteristics of studied patients, and abdominal body fat distribution at baseline and after 8 weeks of 25 g/day of chia consumption

		***n*** **= 25**	
	**Basal**	**Post-chia**	P*
Age (years)	58.0 ± 7.7		
Men/women	15/10		
Adherence (%)	93.0 ± 8.0		
BMI (kg/m^2^)	30.6 ± 3.5	29.9 ± 3.5	< 0.0001
WC (cm)	100.4 ± 9.8	97.9 ± 9.6	< 0.0001
TAF (cm^2^)	538 (433–631)	501 (410–568)	< 0.01
SAF (cm^2^)	317 (255–394)	308 (248–386)	< 0.05
VAF (cm^2^)	188 (164–230)	180 (150–234)	< 0.05

Data are expressed as mean ± standard deviation or median (interquartile range). BMI: body mass index, WC: waist circumference, TAF: total abdominal fat, SAF: subcutaneous abdominal fat, VAF: visceral abdominal fat. *paired Student’s t-test or Wilcoxon matched-pairs signed-rank test, as correspond

**Table 2:** Intake of macronutrients and plasma fatty acid composition, at baseline and after 8 weeks of 25 g/day of chia consumption

			***n* = 25**		
	**Basal**		**Post-chia**		P*
**Macro nutrient intake**					
kJ/day	6748 ± 2030		7045 ± 1628		NS
Fat^a^ (%)	26.3 ± 5.6		30.4 ± 4.4		0.001
Carbohydrate^a^ (%)	51.1 ± 7.2		48.8 ± 5.5		NS
Protein^a^ (%)	22.6 ± 4.9		20.8 ± 3.6		NS
Total fiber (g/day)	17.3 (13.8–33.4)		30.2 (21.7–34.6)		< 0.0001
**Plasma fatty acids**	**μM**	**% TFA**	**μM**	**% TFA**	
SFA	3856 (3078–4750)	33.9	3563 (2692–4341)	33.0	< 0.05
MUFA	2760 (2258–3564)	27.4	2635 (1970–3508)	26.3	< 0.05
PUFA ω −3	270 (173–360)	2.8	328 (233–499)	3.6	< 0.05
PUFA ω −6	3776 (3226–4281)	36.0	3631 (2731–4249)	37.0	NS
ω-6/ω-3	13 (11–18)		11 (8–14)		< 0.0001
ALA	80 (52–112)	0.8	145 (104–255)	1.4	< 0.0001

Data are expressed as mean ± standard deviation or median (interquartile range), TFA: total plasma fatty acids, SFA: saturated fatty acids, MUFA: monounsaturated fatty acids, PUFA: polyunsaturated fatty acids, ALA: alpha-linolenic acid, NS: non-significant. ^a^Expressed as percentage of total consumed energy (1 kcal = 4.186 kJ) *paired Student’s t-test or Wilcoxon matched-pairs signed-rank test, as correspond

Changes in the metabolic profile after chia-intervention were modest, with significant reduction in total cholesterol, non-HDL cholesterol, and FFA (Table 3). An additional analysis showed that, in subjects with initial high triglyceride concentrations (TG ≥ 150 mg/dL, *n* = 16), the improvement in FFA (mmol/L) was greater (basal: 0.74 [0.59–0.85], post-chia: 0.57 [0.47–0.64]; *P* = 0.02). Regarding liver function tests, there were no differences in the median concentrations (Table 3), or in the prevalence of abnormal values (data not shown) after chia supplementation.

**Table 3:** Biochemical parameters at baseline and after 8 weeks of 25 g/day of chia consumption

	***n* = 25**		
	**Basal**	**Post-chia**	P*
TC (mmol/L)	4.8 (4.3–5.2)	4.6 (4.2–4.6)	< 0.05
LDL-C (mmol/L)	2.9 (2.6–3.3)	2.9 (2.5–3.3)	NS
Non-HDL-C (mmol/L)	3.9 (3.2–4.2)	3.7 (3.0–4.1)	< 0.05
HDL-C (mmol/L)	0.93 (0.77–1.10)	0.95 (0.83–1.07)	NS
TG (mmol/L)	1.9 (1.2–2.6)	1.6 (1.1–2.2)	NS
TG/C-HDL	5.2 (2.7–6.9)	3.4 (2.6–6.6)	NS
FFA (mmol/L)	0.68 (0.56–0.80)	0.64 (0.48–0.68)	< 0.05
UA (mmol/L)	0.42 (0.35–0.46)	0.40 (0.34–0.48)	NS
GGT (UI/L)	35 (23–50)	32 (22–46)	NS
AST (UI/L)	23.5 (21–27)	23.7 (20–28)	NS
ALT (UI/L)	29.2 (21–37)	26.9 (20–36)	NS
AST/ALT	0.89 (0.73–0.95)	0.85 (0.68–1.0)	NS

Data are expressed as median (interquartile range), TC: total cholesterol, LDL-C: low density lipoprotein cholesterol, non-HDL-C: non-high density lipoprotein cholesterol, HDL-C: high density lipoprotein cholesterol, TG: triglycerides, FFA: free fatty acids, UA: uric acid, GGT: gamma glutamyl transpeptidase, AST: aspartate aminotransferase, ALT: alanine aminotransferase, NS: non-significant. *Wilcoxon matched-pairs signed-rank test

**Fig. 2:**
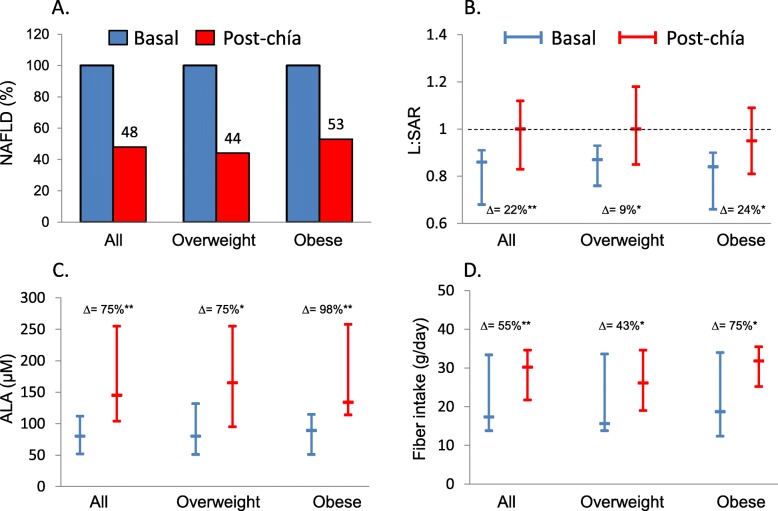
Effects of chia supplementation on non-alcoholic fatty liver disease (NAFLD) prevalence (**a**), liver:spleen attenuation ratio (L:SAR) (**b**), plasma ALA (**c**), and dietary fiber intake (**d**). NAFLD was defined as L:SAR < 1.0, Δ represents percent change between basal and post-chia measurements. L:SAR ratio, ALA, and dietary fiber intake are expressed as median (interquartile range), *P < 0.01, **P < 0.001 (Wilcoxon matched-pairs signed-rank test)

After 8 weeks of chia intervention, a significant body weight loss was observed (median = − 1.4%), with consequent BMI and waist circumference reduction (Table 1). The analysis of the abdominal fat distribution revealed a reduction in TAF, principally due to a greater loss of VAF (− 9%) respect to the SAF deposit (− 5%). Furthermore, 52% of patients showed regression of the NAFLD (Fig. 2, panel A), with an increase of 22% in the liver:spleen attenuation ratio (Fig. 2, panel B). The improvement in the attenuation index was more marked in obese patients (24%) as compared with those with overweight (9%). This amelioration was concomitant to a higher increase in ALA and fiber consumption (panel C and D).

## Discussion

The present study evaluated the effects on NAFLD of an isocaloric diet supplemented with 25 g/day of milled chia. To the best of our knowledge, the present study shows for the first time that milled chia reversed NAFLD in 52% of the treated patients. This was accompanied by decreases in BMI, waist circumference, and VAF reduction, particularly in obese subjects. These results suggest that milled chia intake could be an accessible therapeutic alternative for the control and prevention of NAFLD, and its main causes of morbidity and mortality (T2DM and CAD).

Intervention studies with chia in humans are scarce and vary in the way of seed administration (whole seed soaked, milled, or incorporated into bread), duration of the intervention (weeks or months), type of basal diet (with or without caloric restriction), and characteristics of the included patients (apparently healthy or with diabetes) [14, 20–23]; besides, only few studies have analyzed plasma ALA increase after chia administration [14, 20]. Altogether these factors have given rise to controversies on possible chia beneficial effects. Nieman et al. proved the effect of whole [20] and milled chia seeds [14] on plasma ALA concentrations, finding that, independently of body weight, plasma ALA was increased by 138% when supplementing the habitual diet with 25 g/day of milled chia during 7 weeks [14]; whereas only 24% was increased when the whole seed soaked in water was consumed [20]. Considering these antecedents, in the present study, 25 g/day of milled chia was administered for 8 weeks. In line with Nieman et al., a 75% (40–125%) increase in ALA was reported. However, in contrast to the current and previous results [21, 22], Nieman et al. did not observe anthropometric or metabolic benefits after chia treatment [14, 20]. These inconsistencies could be explained by the clinical and metabolic characteristics of the study subjects. It is possible that the beneficial effects of chia are only evident when milled seed is administered to metabolically abnormal subjects, as Tavares-Toscano et al. reported [22].

In line with previous reports [21, 22], a significant increase in fiber (mean = 10 g/day) was found in the present study. It has been suggested that fiber forms a mechanical barrier in the gut that interferes with glucose and FFA absorption, in turn, promoting an increase in intraluminal viscosity, delayed intestinal transit, and increased production of the glucagon-like peptide-1 (GLP-1), favoring the sensation of satiety [23, 24]. These evidences support the weight loss observed in this and previous studies [22, 23]. Furthermore, the present results provide additional information by showing a reduction in VAF, which is considered a relevant fat deposit due to its association with insulin resistance and other cardiometabolic risk factors [25].

Previously, it had been suggested that a high omega-6:omega-3 index could be implicated in the etiopathogenesis of NAFLD [4, 26]. Evaluation of a dietary pattern study showed that patients with NAFLD had a high consumption of omega-6 polyunsaturated fatty acids [27]. Moreover, in animal models, reduction in the omega-6:omega-3 index decreases the intra-hepatic fat content [4, 28], independently of the bio-conversion of ALA to EPA and DHA [29]. These findings support the current observations regarding a significant reduction in the omega-6:omega-3 index, accompanied by an amelioration in the liver:spleen attenuation ratio. These reductions are similar to those observed in a previous study using a Mediterranean diet [5]. Evidences in animal models suggest that the mechanisms involved in this NAFLD improvement include the modulating effect of ALA on the nuclear receptors SRBP-1c (sterols regulator) and the peroxisomes proliferator activated receptor alpha (PPARα), which modulate de novo lipogenesis and beta-oxidation [9–11].

Therapeutic use of omega-3 fatty acids for the management of NAFLD has gained interest, due to its potential antioxidant effect, among other benefits, since oxidation is part of the pathophysiology of fatty liver disease [3]. Moreover, a meta-analysis showed that dietary supplementation with animal sources of omega-3 fatty acids should be accompanied by antioxidants consumption, such as vitamin E, as this kind of fatty acids are sensitive to oxidation. Additionally, a similar improvement of total antioxidant capacity was observed with plant sources of omega-3 fatty acids [30]. The beneficial effects of omega 3-rich seeds may be explained by their high antioxidants content. Particularly, chia seeds are rich in polyphenols and vitamin E, molecules with a high antioxidant capacity [31]. Insulin-resistance and NAFLD animal model studies have shown that the administration of a chia-based diet, aside from decreasing de novo lipogenesis and improving beta-oxidation [9–11], restores the activity of some enzymes, including catalase and superoxide dismutase, that decrease the hepatic production of some oxidation and inflammation markers [32]. Hence, based on the present and previous studies, it is plausible to hypothesize that these mechanisms could act synergistically to reverse NAFLD. However, further studies are needed.

NAFLD has a multifactorial origin and a certain genetic susceptibility has been reported [2, 3]. Compared with carriers of the I148 allele, M148 allele carriers of the gene PNPLA3 have a greater susceptibility to develop NAFLD [2]. Studies evaluating both, the contribution of PNPLA3 polymorphism (I148M/ PNPLA3 rs738409) and therapeutic strategies on NAFLD are scarce. Nevertheless, it has been suggested that carriers of the I148 allele lose a greater proportion of hepatic fat in response to dipeptidyl peptidase-4 (DPP-4) inhibitors; whereas carriers of the M148 allele are more responsive to the effect of omega-3 fatty acids [33]. Chia supplementation offers a unique opportunity for NAFLD treatment, because in addition to its high omega-3 content, it could increase GLP-1 due to its high viscosity fiber content [23]. The PNPLA3 polymorphism gene has been previously described in the GEA population [34]. The present study included three I/I, eleven I/M, and eleven M/M genotyped subjects, an additional analyses showed that basal hepatic fat content (I/I homozygote: 0.78, I/M heterozygote: 0.89, and M/M homozygote: 0.84) and liver:spleen attenuation ratio improvement (22, 13, and 24%; respectively) were similar among different polymorphisms, suggesting that chia exerted a similar beneficial effect on the whole spectrum of NAFLD patients.

The effect of chia on glucose and lipid levels has been less studied. Nieman et al. did not observe improvements in these parameters when analyzing normolipidemic women [20]. However, Tavares-Toscano et al. [22] found improvements in the metabolic profile, but only in patients with abnormal biochemical parameters. In those subjects, a significant reduction occurred in TC and TG, as well as an increase in HDL-C levels. Our data showed a significant reduction in TC and non-HDL-C, and a non-statistically significant decrease in TG levels. FFA were also significantly reduced, particularly among subjects with initial high TG concentration (> 150 mg/dL). These findings suggest an improvement in adipose tissue insulin sensitivity and reduction in de novo lipogenesis as a result of chia intake.

### Strengths and limitations

The main strength of the present study was the tomographic analysis of abdominal fat, which allowed assessing chia’s effect beyond body weight and its effect on fat depots with a greater pathophysiological transcendence. Based on previous studies, an adequate dose of the milled seed was administered, which allowed for a greater bioavailability of its nutrients and maximal increment in ALA; besides, adherence to treatment was higher than 90%, and this was verified by quantifying ALA in plasma.

The most important limitation was the lack of a control group; however, previous studies agree about the difficulty of having an adequate placebo. In a placebo-controlled chia study, authors analyzed the blinded design in participants, finding that 60% of subjects on the placebo arm acknowledged being on placebo treatment [20]. On the other hand, controlled studies have used a fiber source as placebo, despite that it has not been elucidated whether ALA and fiber exert independent or synergistic beneficial effects. Studies with a cross-over design could be appropriate to confirm the present results. To avoid gastrointestinal adverse events, consumption of milled chia was recommended to occur from breakfast to lunch (always before 6:00 p.m); however, lipid absorption and metabolism may be higher during the beginning of the active/awake period [35]. Hence, a balance between these issues should be considered in further studies. Finally, an additional limitation is that NAFLD improvement was estimated through tomography. Although hepatic biopsy is the gold standard for this measurement, tomography has been shown to be a method that allows identifying intrahepatic fat deposits higher than than 30%, with a sensitivity and specificity of 80% [36].

## Conclusions

The results of the present study show that 25 g/day of milled chia ameliorates NAFLD. Due to its composition, chia could be an accessible vegetal source of omega-3 fatty acids, antioxidants, and fiber. These phytochemicals have the potential to prevent the appearance of metabolic abnormalities, advanced stages of NAFLD, and development of T2DM and CAD in NAFLD patients. Functional foods are suggested as part of innovative therapies for the treatment of cardiometabolic diseases. Although findings of the present study expand this information, more studies are needed to consolidate these nutriments as therapeutical alternatives.

## Data Availability

According to the policy of BMC, we would like to inform that all the datasets analyzed during the current study are available from the corresponding author upon reasonable request.

## Abbreviations

NAFLD: non-alcoholic fatty liver disease
ALA: alpha linolenic acid
EPA: eicosapentaenoic acid
DHA: docosahexaenoic acid
BMI: body mass index
VAF: visceral abdominal fat
SAF: subcutaneous abdominal fat
TAF: total abdominal fat
L:SAR: liver:spleen attenuation ratio
FFA: free fatty acids
T2DM: type 2 diabetes mellitus
CAD: coronary artery disease
HOMA-IR: homeostasis model assessment for insulin resistance
GEA: Genetics of the Atherosclerotic Disease study
